# A new prognostic score for predicting survival in patients treated with robotic stereotactic radiotherapy for brain metastases

**DOI:** 10.1038/s41598-021-98847-3

**Published:** 2021-10-13

**Authors:** Magdalena Stankiewicz, Bartlomiej Tomasik, Slawomir Blamek

**Affiliations:** 1 Brachytherapy Department, Maria Sklodowska-Curie National Research Institute of Oncology, Gliwice Branch, Gliwice, Poland; 2 Radiotherapy Department, Maria Sklodowska-Curie National Research Institute of Oncology, Gliwice Branch, Gliwice, Poland; 3 Department of Biostatistics and Translational Medicine, Medical University of Lodz, Lodz, Poland; 4 Department of Radiation Oncology, Dana-Farber Cancer Institute, Boston, MA USA

**Keywords:** Oncology, Metastasis

## Abstract

The study aimed to analyze potential prognostic factors in patients treated with robotic radiosurgery for brain metastases irrespective of primary tumor location and create a simple prognostic score that can be used without a full diagnostic workup. A retrospective analysis of 142 patients with 1–9 brain metastases treated with stereotactic radiosurgery (1–4 fractions) was performed. Volumes of all lesions were calculated using linear dimensions of the tumors (CC, LR, AP) and 4/3*π*(CC/2)*(LR/2)*(AP/2) formula. Kaplan–Meier method and log-rank test were used to analyze survival. Variables significantly associated with overall survival in univariate analysis were included in Cox multivariate analysis. The validity of the model was tested with the bootstrap method. Variables from the final model were used to construct a new prognostic index by assigning points according to the impact of a specific variable on overall survival. In the multivariate analysis, four factors: Karnofsky Performance Status (p = 0.000068), number of brain metastases (p = 0.019), volume of the largest lesion (p = 0.0037), and presence of extracerebral metastases (p = 0.0017), were independent predictors of survival. Total scores ranged from 0 to 12 points, and patients were divided into four groups based on median survival of each subgroup: 0–1 points—18.8 months, 2–3 points—16.9 months, 4–5 points—5.6 months, and ≥ 6 points—4.9 months (p < 0.001). The new prognostic index is simple to calculate. It has a strong prognostic value in a heterogeneous population of patients with a various number of brain metastases, but its value requires confirmation in another cohort.

## Introduction

Brain metastases (BMs) occur in 9–40% of all cancer patients during the course of their disease. The reported incidence of metastatic brain tumors is increasing^1^. Therapeutic options for patients diagnosed with metastatic brain tumors include neurosurgical resection, whole brain radiotherapy (WBRT), stereotactic radiosurgery (SRS), best supportive care and a combination of these methods. Treatment selection strongly depends on pretreatment factors, prognosis and patients’ treatment preferences. Quickly, it has become clear that not all patients with BMs have the same prognosis. There is a subset of patients who may live for years and benefit from more aggressive therapies. In order to choose an appropriate treatment regimen, personalized approaches are required. Therefore, a useful prognostic score that helps predict survival is essential to guide treatment decisions for an individual patient and properly stratify patients in future research. Numerous prognostic indices have been proposed: Recursive Partitioning Analysis (RPA), Score Index for Radiosurgery (SIR), Basic Score for Brain Metastases (BSBM), Golden Grading System (GGS), Graded Prognostic Assessment (GPA), Diagnosis-specific Graded Prognostic Assessment (ds-GPA) and Rades score (Table 1)^2–11^. All of them have identified Karnofsky Performance Status (KPS) as the most important prognostic factor. The other factors are systemic disease status, size and number of brain metastases, age and primary tumor location. Nevertheless, all previously published indices have some limitations:

- RPA, BSBM and GGS do not take the number of brain metastases into account, whereas this parameter has an established prognostic value,
- RPA, SIR and BSBM require the assessment of systemic disease status, which may be difficult to achieve before the introduction of BM treatment,
- SIR requires a volume of the largest BM, which is usually available after a decision concerning treatment was made and requires time-consuming contouring,
- ds-GPA requires detailed diagnostics with complex pathological tests, not always available at the time of clinical decision making.

**Table 1:** Published prognostic indices.

**Recursive partitioning analysis (RPA)**					
Class I	Age < 65 years, KPS ≥ 70, controlled primary tumor, no ECM				
Class II	All patients not in Class I or III				
Class III	KPS < 70				
**Score index for radiosurgery (SIR)**					
Score	0	1	2		
Age (years)	≥ 60	51–59	≤ 50		
KPS	≤ 50	60–70	80–100		
Systemic disease	PD	SD	CR or NED		
Number of BMs	≥ 3	2	1		
Volume of the largest lesion (ml)	> 13	5–13	< 5		
**Basic score for brain metastases (BSBM)**					
Score	0	1			
KPS	50–70	80–100			
Control of primary tumor	No	Yes			
ECM	Present	None			
**Golden grading system (GGS)**					
Score	1	0			
Age ≥ 65 years	Yes	No			
KPS < 70	Yes	No			
ECM	Present	None			
**Graded prognostic assessment (GPA)**					
Score	0	0.5	1		
Age (years)	≥ 60	50–59	< 50		
KPS	< 70	70–80	90–100		
Number of BMs	> 3	2–3	1		
ECM	Present	n/a	None		
**Diagnosis-specific Graded Prognostic Assessment (ds-GPA)**					
*SCLC*					
Score	0	0.5	1		
Age (years)	> 60	50–60	< 50		
KPS	< 70	70–80	90–100		
ECM	Present	n/a	None		
Number of BMs	> 3	2–3	1		
*NSCLC (Lung-molGPA)*					
Age (years)	≥ 70	< 70	n/a		
KPS	< 70	70–80	90–100		
ECM	Present	n/a	None		
Number of BMs	> 4	1–4	n/a		
Gene status	EGFR neg/unk and ALK neg/unk	n/a	EGFR pos or ALK pos		
*Melanoma/RCC*					
Score	0	1	2		
KPS	< 70	70–80	90–100		
Number of BMs	> 3	2–3	1		
*Breast cancer*					
Score	0	0.5	1	1.5	2
Age (years)	> 60	< 60	n/a	n/a	n/a
KPS	≤ 50	60	70–80	90–100	n/a
Subtype	Basal	n/a	Luminal A	HER2	Luminal B
*GI cancer*					
Score	0	1	2	3	4
KPS	< 70	70	80	90	100
**Rades score**					
		Score			
Age	≤ 60 years	5			
	> 60 years	4			
KPS	> 70	7			
	70	5			
	< 70	1			
ECM	None	6			
	Present	3			
Number of BMs	1	7			
	2–3	6			
	≥ 4	3			
Interval from tumor diagnosis to WBRT	> 6 months	5			
	≤ 6 months	4			

*KPS* Karnofsky Performance Status, *BMs* brain metastases, *PD* progressive disease, *SD* stable disease, *CR* complete remission, *NED* no evidence of disease, *ECM* extracranial metastases, *NSCLC* non-small cell lung cancer, *SCLC* small cell lung cancer, *RCC* renal cell carcinoma, *GI* gastrointestinal, *n/a* not applicable, *neg/unk* negative or unknown, *pos* positive, *WBRT* whole brain radiotherapy.

From validated prognostic indices, only RPA was initially designed for patients treated with WBRT. The SIR, BSBM and GGS were initially designed for patients undergoing stereotactic radiosurgery, whereas others (GPA, ds-GPA and Rades score) were constructed based on the analysis of patients treated with various regimens (surgery, WBRT or SRS).

The objective of this study was to identify independent pretreatment factors associated with overall survival and create an easy-to-use prognostic score for patients with brain metastases irrespective of primary tumor location and for those without full diagnostic workup. Moreover, we aimed to validate previously described stratification systems in the Polish population of patients with brain metastases.

## Methods

The present study is a single-institution retrospective review of 142 consecutive patients treated with stereotactic radiotherapy for brain metastases between the years 2011 and 2015. All methods were carried out in accordance with relevant guidelines and regulations. The experimental protocol was approved by Maria Sklodowska-Curie National Research Institute of Oncology Bioethics Committee, and a waiver on informed consent was obtained from the aforementioned committee (KB/430-05/21). The eligibility criteria included patients in good general condition (KPS ≥ 70), without leptomeningeal disease, who were not eligible for surgery or refused invasive treatment. All SRS procedures were performed on the CyberKnife accelerator (CK) (Accuray Incorporated, Sunnyvale, California, United States). The group consisted of 51 (36%) men and 91 (64%) women. Mean age was 58 years (range 29–84 years). In 53 patients (37.3%) the primary tumor was lung cancer, in 37 (26.1%)—breast cancer, in 12 (8.5%)—kidney cancer, in 9 (6.35%)—melanoma, and in 9 (6.35%)—colorectal cancer. In five patients (4%) the location of the primary tumor could not be determined (CUP—cancer of unknown primary). Treatment of brain metastases using stereotactic radiotherapy was performed at least twice in 39 patients. The mean time from the primary diagnosis to the diagnosis of brain metastases was 39 months, median—24 months. In 10% of patients cerebral dissemination was diagnosed before the primary tumor was detected. The time from primary diagnosis to the first CK treatment of brain metastases ranged from 0 to 256 months (mean—40 months, median—24 months). Nearly 4% of patients underwent stereotactic treatment of BMs before the primary tumor was diagnosed. The mean time between the diagnosis of brain metastases and CK radiosurgery was 6.9 months, median—2.5 months. Whole brain radiation therapy was carried out in 70% of patients (in 51.5% before, in 15.5% after CK treatment, and in 3% WBRT was used twice—before CK and as a form of salvage treatment due to progression after stereotactic radiosurgery). In 48% of patients systemic therapy (chemo-, hormone- or immunotherapy) was additionally used. The total number of irradiated lesions was 270. The maximum number of brain metastases treated in one patient was 9 (mean—2 lesions, median—1 lesion). In 55.6% of cases a single lesion was irradiated, in 21.8%—2 lesions, and in 22.6%—3 or more lesions. Extracranial metastases (ECM) were diagnosed in 62.7% of patients. Progressive disease (PD) evaluated within 2 months before BMs treatment was diagnosed in 45% of cases, stable disease (SD) in 30.3% and complete remission (CR) in 24.7%. The volume of the largest lesion ranged from 0.02 to 47.65 ml (mean—9.41 ml, median—5.15 ml). The total tumor volume (TTV) was defined as the volume of all brain metastases and ranged from 0.06 to 63.96 ml (mean—10.29 ml, median—5.34 ml). In patients with multiple BMs the volume of the largest tumor comprised 25.1–99% of the TTV (mean—71.4%, median—72.7%). Volumes of all lesions were calculated using linear dimensions of the tumors (CC, LR, AP) obtained from pretreatment imaging tests. Considering the fact that brain metastases usually have a sphere-like shape, the formula for the volume of a spheroid: 4/3*π*(CC/2)*(LR/2)*(AP/2) was used to simplify volume assessment. The overall survival (OS) was defined as the time between the last fraction of stereotactic radiosurgery and the last visit in our Institute (censored at this time) or the patient's death. Survival times were analyzed depending on parameters with known or potential prognostic and predictive value: number and volume of BMs, TTV, ECM, control of the primary tumor, location and pathology of the primary tumor, status of the systemic disease, doses and number of fractions, time intervals between primary diagnosis and BMs diagnosis, as well as between BMs diagnosis and CK radiosurgery. Single or multiple fractions were used depending on the volume and location of brain metastasis. According to the treatment protocol from our Institute, the SRS doses corresponded to the doses used in RTOG 90-05 study^12^. Single-dose stereotactic radiosurgery was used in 48.6% of cases with doses ranging from 5 to 24 Gy (mean—16.6 Gy, median—18 Gy). The one patient irradiated with a single dose of 5 Gy was initially intended to receive a fractionated schedule, but the treatment was terminated after the first fraction. In fractionated regimen, doses per fraction ranged between 5 and 13 Gy (mean—8.2 Gy, median—8 Gy), whereas the total doses ranged from 12 to 30 Gy (mean—19.5 Gy, median—19 Gy).

Statistical analysis was performed using the Statistica 13.1 (StatSoft Incorporated, Tulsa, Oklahoma, United States), R (version 4.1.0, R Foundation for Statistical Computing, Vienna, Austria) and RMS package. Kaplan–Meier estimator and log-rank test were used to analyze survival. Variables significantly associated with overall survival in univariate analysis were included in Cox multivariate analysis. The validity of the model was tested with the bootstrap method. Variables from the final model were used to construct a new prognostic index by assigning points according to the impact of a specific variable on overall survival. The *p* value < 0.05 was considered statistically significant. Prognostic factors for survival identified with Cox multivariate were used to develop the nomograms for early death (< 3 months) and long-term survival (> 1 year) prediction. The areas under the curve (AUCs), obtained using receiver operating characteristics (ROC), of the developed models were compared with the value of AUCs described elsewhere^13^. ROC curves were compared using DeLong’s test.

## Results

Median follow-up was 38.2 months (range 0–67.8 months). Median overall survival was 8 months. The 6-, 12- and 24-month overall survival rates were 58%, 39.3% and 19.7%, respectively. Factors significantly associated with overall survival in univariate analysis were as follows: KPS, number of brain metastases (single vs. multiple), volume of the largest lesion, total dose, TTV, ECM and control of the primary tumor. There was no significant difference in OS between patients with two, three or > 3 metastases (p = 0.26). Age, gender, status of systemic disease, fractionation scheme, application of WBRT or systemic treatment were not statistically significant predictors of survival in the whole cohort (Table 2). Analysis of the association between WBRT and overall survival according to the number of BMs showed that the best OS was observed in the subgroup of patients with a single lesion, who underwent WBRT, while the worst in patients with multiple lesions, who underwent WBRT—these differences were statistically significant (p = 0.037). There was no association between time intervals (from primary diagnosis to BMs diagnosis and from BMs diagnosis to CK treatment) and overall survival (p = 0.16 and p = 0.14, respectively). The prognostic value of all previously described indices was confirmed in the population of patients treated in our center (Table 3). Repeat radiosurgical treatment was associated with better OS.

**Table 2:** The univariate analysis for survival.

Variable	*p*
KPS (70 vs. 80 vs. 90–100)	**0.00063**
Number of BMs (single vs. multiple)	**0.013**
Volume of the largest lesion (> 5 ml vs. ≤ 5 ml)	**0.018**
TTV (> 5 ml vs. ≤ 5 ml)	**0.0066**
Age (65 years vs. ≤ 65 years)	0.43
Gender (male vs. female)	0.45
Primary tumor location	0.76
Systemic disease (PD vs. SD vs. CR)	0.11
ECM (present vs. absent)	**0.0085**
Control of primary tumor (yes vs. no)	**0.042**
WBRT (yes vs. no)	0.67
Systemic treatment (yes vs. no)	0.87
Fractionation (single vs. multiple fractions)	0.41
Total dose (> 18 Gy vs. ≤ 18 Gy)	**0.0014**
Repeat SRS (yes vs. no)	**< 0.00000**

*KPS* Karnofsky Performance Status, *BMs* brain metastases, *TTV* total tumor volume, *PD* progressive disease, *SD* stable disease, *CR* complete remission, *ECM* extracranial metastases, *WBRT* whole brain radiotherapy, *SRS* stereotactic radiosurgery. Figures marked in bold indicate the factors significantly associated with overall survival in univariate analysis.

**Table 3:** The χ^2^ test results for prognostic indices.

Prognostic index	*p*
RPA	0.0022
SIR	0.00021
BSBM	0.0014
GGS	0.0011
GPA	0.00047
Ds-GPA	0.0047
RADES	0.0034
CPI	0.00033

*RPA* Recursive Partitioning Analysis, *SIR* Score Index for Radiosurgery, *BSBM* Basic Score for Brain Metastases, *GGS* Golden Grading System, *GPA* Graded Prognostic Assessment, *ds-GPA* diagnosis-specific Graded Prognostic Assessment.

**Figure 1:**
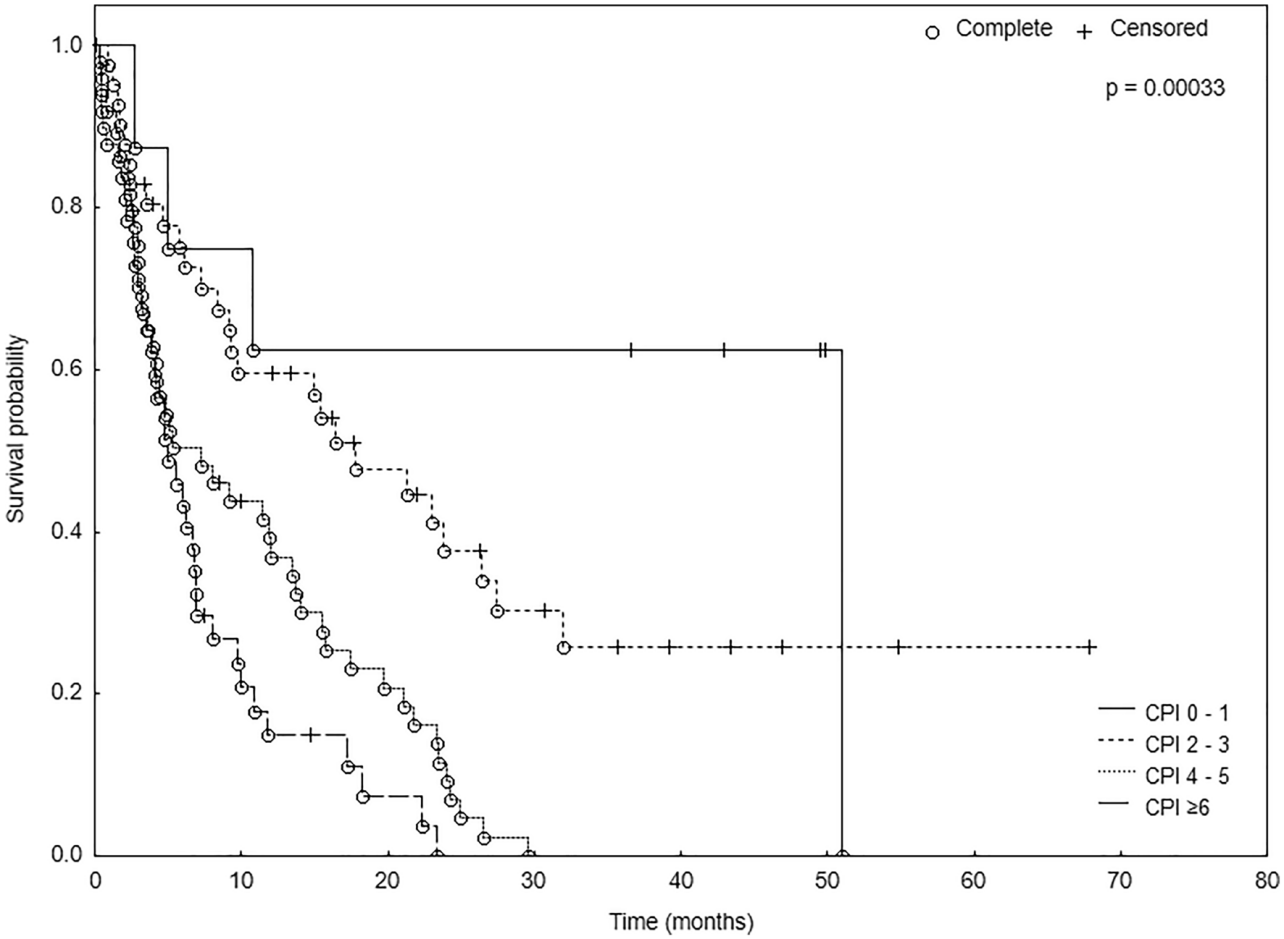
Kaplan–Meier curves for OS according to CPI.

In the multivariate analysis, four factors: KPS, number of BMs, volume of the largest lesion and ECM, were independent predictors of survival (Table 4). The analyzed population was subjected to re-sampling with the bootstrap method, which resulted in obtaining very similar estimations of the model parameters (Table 5). Consequently, these four variables were incorporated in the new prognostic score—Comprehensive Prognostic Index (CPI). The corresponding scoring points of the variables are summarized in Table 6. The resulting score values range between 0 and 12. Patients were divided into four groups based on the median survival of each subgroup. Median OS was 18.8 months for patients with 0–1 points, 16.9 months for those with 2–3 points, 5.6 months for ones with 4–5 points, and 4.9 months for patients with ≥ 6 points (p < 0.001, Fig. 1).

**Table 4:** The multivariate Cox analysis for survival.

Variable	*p*	HR	Low 95% CI	High 95% CI
KPS (70 vs. 80 vs. 90–100)	0.000068	0.96	0.93	0.98
Number of BMs (single vs. multiple)	0.019	1.19	1.03	1.37
Volume of the largest lesion (> 5 ml vs. ≤ 5 ml)	0.0037	1.02	1.01	1.04
ECM (present vs. none)	0.0017	2.11	1.4	3.19

*KPS* Karnofsky Performance Status, *BMs* brain metastases, *ECM* extracranial metastases, *HR* hazard ratio, *CI* confidence interval.

**Table 5:** The bootstrap analysis based on 1000 resamples.

	Value	Bootstrap average	Bootstrap standard error	Bootstrap low 95% CI	Bootstrap high 95% CI
KPS	− 0.0465	− 0.0473	0.0123	− 0.0732	− 0.0239
Number of BMs	0.1727	0.1682	0.0655	0.0382	0.2987
Volume of the largest lesion	0.0244	0.0246	0.0073	0.0113	0.0399
ECM	0.3743	0.3869	0.1048	0.1902	0.6012
R2	0.3025	0.3119	0.0725	0.1760	0.4496
AIC	889.2463	888.566	43.0583	799.4513	970.3159

*KPS* Karnofsky Performance Status, *BMs* brain metastases, *ECM* extracranial metastases, *AIC* Akaike Information Criterion, *CI* confidence interval.

**Table 6:** Comprehensive prognostic index (CPI).

Points	0	1	2	3	4
KPS	100	90	80	70	≤ 60
Number of BMs	1	2	3–6	≥ 7	–
Volume of the largest lesion	< 10 cm^3^	10–15 cm^3^	15–35 cm^3^	> 35 cm^3^	–
ECM	None	–	Present	–	–

*KPS* Karnofsky Performance Status, *BMs* brain metastases, *ECM* extracranial metastases.

**Figure 2:**
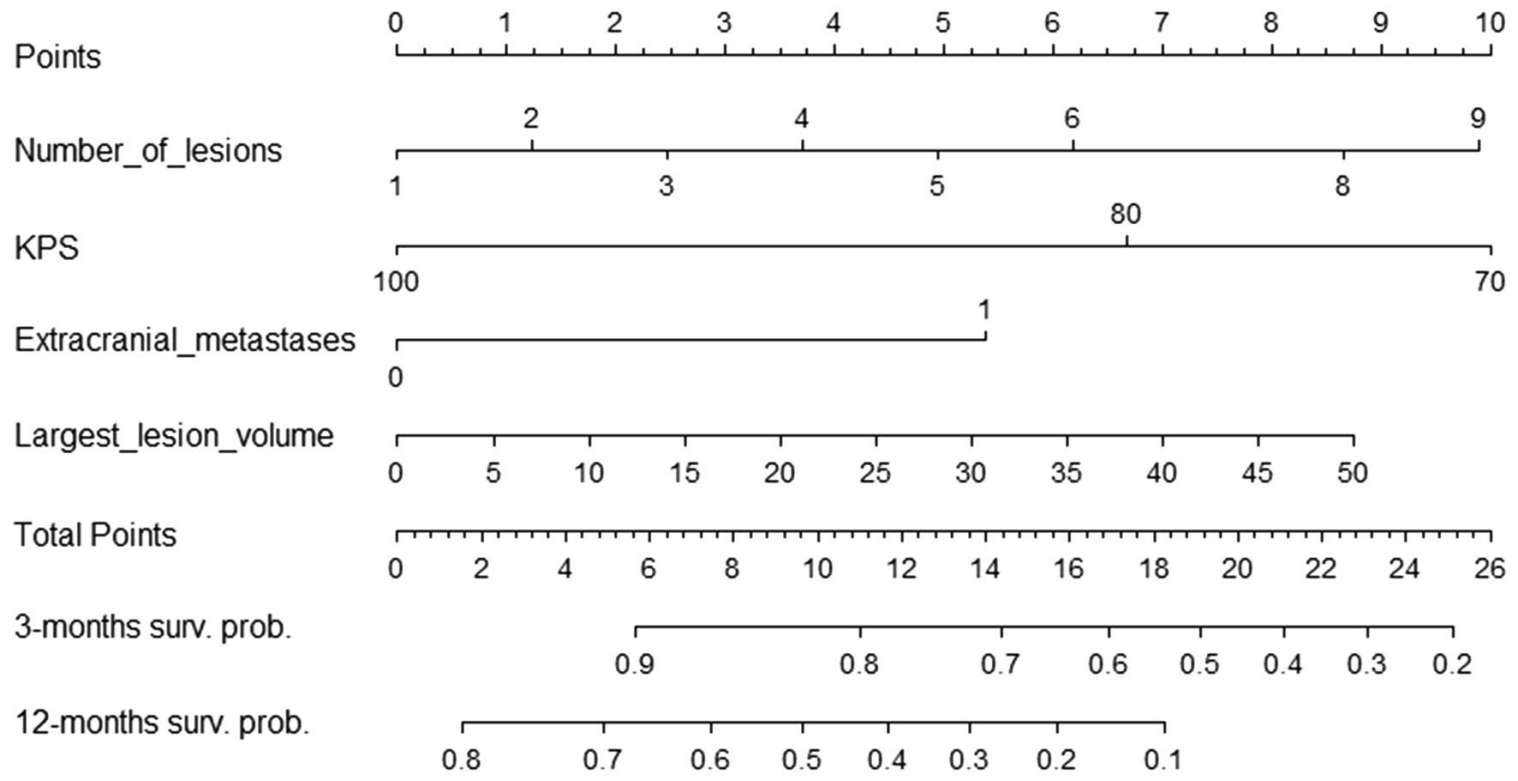
Nomogram for prediction of survival based on the outcome of 142 patients treated with SRS alone for brain metastases.

**Figure 3:**
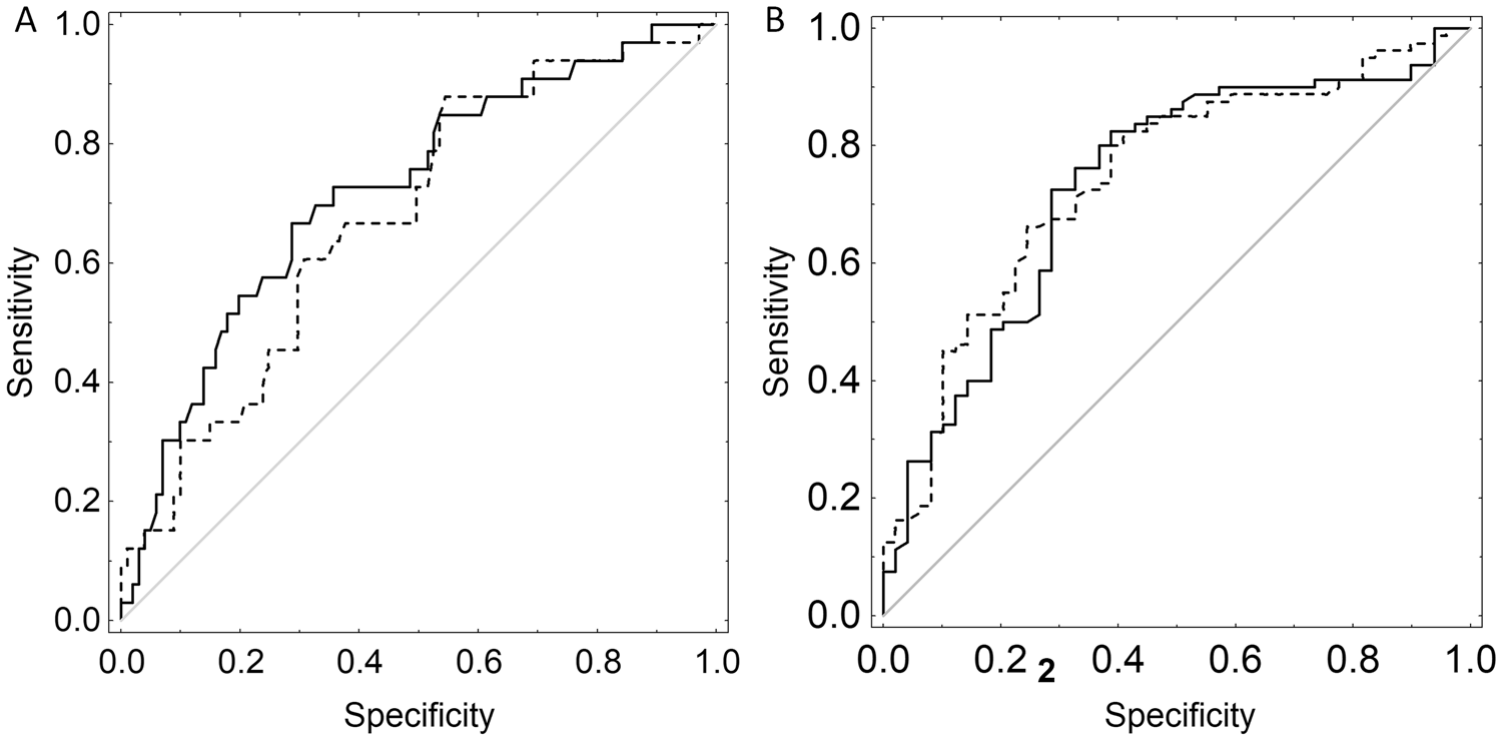
ROC analysis of the nomogram for the prediction of (**A**) short-term (3 months) and (**B**) long-term survival after SRS alone for brain metastases in both the Gliwice (dashed line) and the Dutch Radiation Oncology centers (solid line) cohorts.

The nomograms specific for the prediction of early death and long-term survival are presented in Fig. 2. The results of AUCs in ROC analysis comparison between our nomograms and the prognostic models from Dutch centers are presented in Fig. 3 and Table 7.

**Table 7:** Accuracy of the prediction of early (< 3 months) and long-term survival (> 12 months) of the proposed nomogram compared to the nomogram of the Dutch Radiation Oncology centers.

Center	AUC (95% CI)	*p* value
**Early death prediction (< 3 months)**		
Gliwice	0.68 (0.58–0.78)	0.549
Dutch radiation oncology centers	0.72 (0.62–0.82)	
**Long-term survival prediction (> 12 months)**		
Gliwice	0.74 (0.66–0.83)	0.877
Dutch radiation oncology centers	0.73 (0.64–0.83)	

## Discussion

Despite extensive research on brain metastases treatment and the use of radiosurgery techniques, many issues are still doubtful. In order to optimize personalized treatment approach in a single patient, an accurate estimation of the patient’s prognosis is essential. For that reason, prognostic and predictive tools are needed. The newly proposed stratification system was effective in identifying patients with different outcomes in an easy way. The variables included in CPI have been described in most studies as the most important prognostic factors for survival but were never combined in a single prognostic index^2–11^. The newly designed index has several advantages. The set of variables included in the model is very helpful for the initial assessment of patient’s prognosis. Moreover, it does not require the primary tumor diagnosis or pathological examination and therefore may be implemented in patients without full diagnostic workup. The general condition is assessed routinely, extracranial dissemination is generally known when radiosurgical treatment is considered, and the remaining are known from standard MRI, which should be performed before treatment decision making. Thanks to the simplified method of the tumor volume calculation, the MRI examination does not require specialized volumetric analysis and contouring of the tumor, and is independent of the volume calculation algorithm inherent for the certain treatment planning system. Due to the incorporation of the volume of the largest lesion exclusively, there is no need to perform calculations for all metastases. Consequently, CPI is simple to use, does not require complex tests and is potentially suitable for all patients regardless of the diagnosis. However, its value needs to be verified in other groups of patients from various centers, as well as in sufficiently numerous groups with different types of cancer.

Despite the heterogeneity of our series, especially concerning the primary tumor location, systemic disease status and various types of applied treatment regimens, all tested stratification scores were helpful in the prognostication of survival. The SIR and CPI proved to be the most reliable predictors of OS. The values of the designed nomograms were comparable to those described by Zindler et al.^13^. The Dutch nomogram predicted early death slightly better, while our was superior in 1-year survival prediction. These differences were not statistically significant (p = 0.549 and p = 0.877, respectively). However, it should be noted that patients in these studies differ significantly. Our group is highly heterogeneous with various primary tumors, characterized by a different clinical course, biology and systemic treatment options. Whereas in the Dutch study, all patients were diagnosed with non-small cell lung cancer.

Among all available prognostic indices, SIR and BSBM were designed based on small groups of patients with BMs (34 and 110, respectively). Nevertheless, they are widely used and help in treatment decision making. The ds-GPA is probably the most commonly used stratification system in patients with brain metastases, as it can predict survival most accurately. However, it can only be implemented in patients with selected primary tumors and full diagnostic workup. In those with CUP or without full diagnostic workup, this index is useless.

The prognosis in patients with multiple BMs changes with the number of lesions, but this effect, although significant enough to be included in the model, is less relevant than other factors included in the index. Clearly, better survival was observed in patients with a single brain lesion. Our results are consistent with those of Yamamoto et al. prospective study^14^. However, it is believed that the data indicating a similar prognosis in patients with 2–4 and 5–10 metastases undergoing SRS cannot be simply generalized in the European or American population. This is because of the known differences in the molecular characteristics of tumors in Japan and Europe or the USA^15–17^. However, the results of our analysis indicate that, despite the presumably different molecular characteristics of the group, the prognosis of patients with numerous metastases is similar to the prognosis of patients with two lesions and does not tend to differ much from the Japanese population.

The results of our analysis also confirm a high value of hypofractionated stereotactic radiotherapy. Overall survival of patients treated with this method was similar to OS in those treated with a single dose of radiation therapy. In 2011 Kim et al. published the results of the first retrospective comparative analysis of fractionated and single-dose stereotactic radiotherapy in the treatment of brain metastases. Adverse prognostic factors (such as the presence of ECM or previous WBRT) were significantly more frequent in patients treated with fractionated regimens (p < 0.01 and p = 0.04, respectively). Nonetheless, OS was not related to the used fractionation scheme (p = 0.89)^18^. This demonstrates the high potential of hypofractionated stereotactic radiotherapy. Its implementation in patients with the worst prognosis gives results that do not differ much from those obtained using a single fraction in patients with potentially better survival prognosis. A recently published meta-analysis of 24 studies by Lehrer et al. confirmed similar efficacy of multi-fraction and single-dose stereotactic radiosurgery. No differences in local control were observed between different fractionation regimens (p = 0.38), which is in agreement with our results^19^.

Worse survival was observed in patients with a larger TTV or volume of the largest lesion. A Japanese study published in 2009 assessed the effectiveness of stereotactic radiosurgery in treating multiple brain metastases in patients with extrapulmonary primary tumors. This analysis confirmed that both total tumor volume and volume of the largest lesion are statistically significant predictors of survival (p < 0.0001 and p = 0.0003, respectively)^20^. In a retrospective analysis by Susko et al., worse survival was observed in the group of patients with larger TTV (p = 0.031)^21^. In our material, the TTV parameter lost its significance in the multivariate analysis. However, a significant relationship between volume of the largest lesion (strongly correlated with TTV) and OS remained.

In the subgroup of patients with single BM, better OS was observed in those who underwent whole brain irradiation. These patients were in a better general condition than those who were not treated with WBRT. This may suggest that patients in good condition with a single brain metastasis benefit from maximum treatment intensification. Similar results were obtained in a randomized phase III RTOG 95-08 study comparing survival of patients with 1–3 brain lesions undergoing WBRT with those undergoing WBRT and stereotactic boost. Significantly better overall survival was observed in the subgroup of patients with single brain metastasis who received multimodal treatment (p = 0.039). This effect was not observed in patients with multiple brain lesions^22^. The secondary analysis of RTOG 95–08 results showed that patients from the most favorable GPA prognostic subgroup benefit from a combination of whole brain irradiation with SRS, regardless of the number of brain lesions (p = 0.05)^23^. Sneed et al. also analyzed the effect of adding WBRT to stereotactic radiotherapy in the treatment of BMs. No statistically significant differences in OS were observed in the studied subgroups (p = 0.93)^24^. The results of the study by Aoyama et al. indicate that OS in patients with non-small cell lung cancer and brain metastases, in whom WBRT was conducted in addition to SRS, is significantly longer. However, this difference was only noticeable in the subgroup with the best prognosis according to the ds-GPA index (p = 0.04), which is in line with our findings^25^. Due to the excellent prognosis of CPI class I, in these patients the use of aggressive extracranial treatment with ablative intent should be considered. Currently, due to effective salvage treatment, combining SRS with WBRT is no longer routinely indicated. Instead, salvage SRS should be considered in case of failure after primary treatment, which is also currently the standard mode of operation in our center.

It should be noted that the current study has several limitations. First of all, the retrospective character of the analysis is associated with unavoidable bias because of possibly incomplete or inaccurate medical information. Moreover, selection bias inherent in intergroup comparisons in retrospectively analyzed populations could affect the obtained results and hinder their interpretation. The analyzed group is heterogeneous, both with regard to the location of the primary tumor and pathological diagnosis, as well as in the type and sequence of treatment performed, which also impedes the objective interpretation of the results. On the other hand, it is typical for everyday clinical practice, as opposed to carefully selected subjects enrolled into prospective clinical trials. Nevertheless, a retrospective, single-center nature of the study in a limited population does not allow to draw firm general conclusions for the entire population of BMs patients.

## Conclusions

The new prognostic index allows for a simple and reliable assessment of prognosis and could be used for initial prognostication but requires validation in an independent group of patients.

Patients with a single brain metastasis have the best prognosis. In patients with multiple BMs, the prognosis only moderately changes with the number of tumors. Therefore, the number of lesions should not be included in the eligibility criteria for the treatment of brain metastases with stereotactic radiosurgery techniques.

The prognostic indices described in the literature are applicable in the Polish population of patients with cerebral dissemination.
